# Differences in Proprioception Between Young and Middle-Aged Adults With and Without Chronic Low Back Pain

**DOI:** 10.3389/fneur.2020.605787

**Published:** 2020-12-21

**Authors:** Sabina M. Pinto, Jason P. Y. Cheung, Dino Samartzis, Jaro Karppinen, Yong-ping Zheng, Marco Y. C. Pang, Arnold Y. L. Wong

**Affiliations:** ^1^Department of Rehabilitation Sciences, The Hong Kong Polytechnic University, Hong Kong, China; ^2^Department of Orthopedics and Traumatology, The University of Hong Kong, Hong Kong, China; ^3^Department of Orthopedics Surgery, Rush University Medical Center, Chicago, IL, United States; ^4^International Spine Research and Innovation Initiative, Rush University Medical Center, Chicago, IL, United States; ^5^Medical Research Center Oulu, Oulu University Hospital and University of Oulu, Oulu, Finland; ^6^Rehabilitation Services of South Karelia Social and Health Care District, Lappeenranta, Finland; ^7^Department of Biomedical Engineering, The Hong Kong Polytechnic University, Hong Kong, China

**Keywords:** proprioception, proprioceptive reweighting, chronic low back pain, reposition sense, CLBP

## Abstract

**Introduction:** While young adults with chronic low back pain (CLBP) exhibit impaired lumbar proprioception, it remains unclear if the same phenomenon is observed in middle-aged adults with CLBP.

**Objectives:** This study aimed to investigate whether young or middle-aged adults with CLBP displayed different proprioception ability as compared to age-matched asymptomatic controls.

**Methods:** Sixty-four young adults with [median age:34 [interquartile range (IQR): 29–37] years] and without [median age:29 (IQR; 23–34) years] CLBP, and 87 middle-aged adults with [median age:53 (IQR: 49–58) years] and without [median age: 54 (IQR: 45–64) years] CLBP underwent postural sway tests on a force-plate with (unstable surface) and without a foam (stable surface), while bilateral L5/S1 multifidi and triceps-surae were vibrated separately. An individual's proprioception reweighting ability was estimated by relative proprioceptive reweighting (RPW). Higher RPW values indicate less reliance on lumbar multifidus proprioceptive signals for balance. Participants also underwent lumbar repositioning tests in sitting to determine repositioning errors in reproducing target lumbar flexion/extension positions.

**Results:** Young adults with CLBP demonstrated significantly higher median RPW values than age-matched asymptomatic controls for maintaining standing balance [stable surface: CLBP: 0.9 (IQR: 0.7–0.9), asymptomatic: 0.7 (IQR: 0.6–0.8), *p* < 0.05; unstable surface: CLBP: 0.6 (IQR: 0.4–0.8), asymptomatic: 0.5 (IQR: 0.3–0.7), *p* < 0.05]. No significant differences in repositioning error were noted between young or middle-aged adults with and without CLBP (*p* > 0.05). RPW values were unrelated to repositioning errors in all groups (*p* > 0.05).

**Conclusion:** Young adults with CLBP, and middle-aged adults with and without CLBP had inferior proprioceptive reweighting capability. This finding may indicate potential age-related deterioration in central and peripheral processing of lumbar proprioceptive signals. Future studies should use advanced imaging and/or electroencephalogram to determine mechanisms underlying changes in proprioceptive reweighting in middle-aged adults.

## Introduction

Low back pain (LBP) is the leading cause of disability worldwide (1, 2). Over 80% of people may experience LBP at least once in their lifetime. Up to 90% of LBP cases have unknown etiology and are diagnosed with non-specific LBP (3). Although most patients with acute LBP recover spontaneously, ~20% of cases develop chronic LBP (CLBP) (4) that lasts continuously for 3 months or more (5), resulting in disability and high medical costs (6). Importantly, CLBP is more prevalent among middle-aged adults aged 50 years or older (24.8%) (7) as compared to young adults aged 20–30 years (4.2%) (8).

Pain can induce inflammatory response in paraspinal muscles causing transformation of slow twitch muscle fibers to fast twitch fibers, muscle atrophy, and altered muscle function (e.g., proprioception) (9). Altered lumbar proprioception has been found to be a risk factor for the development, maintenance, and/or recurrence of LBP in young adults (10, 11). Proprioception involves conscious and unconscious awareness of joint position sense, kinesthesia, and force sense of body parts without vision (12–14). Since paraspinal muscles contain abundant muscle spindles (15), they play an important role in generating proprioceptive signals to monitor midrange spinal motion (10, 15). Impaired lumbar proprioception may affect the quality of trunk movement, and increase the risk of back injury (16).

Unconscious lumbar proprioception can be assessed by the relative proprioceptive reweighting (RPW) ratio following disturbance in proprioceptive signals in paraspinal and calf muscles with standing without vision (17, 18). Proprioceptive reweighting is a process by which the central nervous system (CNS) alters the weight allocated to proprioceptive signals in different body parts to maintain standing balance (18). Compared to age-matched asymptomatic individuals, young adults aged 18–25 years with CLBP cannot adjust their proprioceptive weighting and rely more on ankle proprioception than back extensor proprioception to maintain standing balance, irrespective of stable/unstable surfaces (11, 17, 19). Conversely, symptomatic older people (average age: 75 years) with spinal column stenosis and spondylitis deformans showed no significant difference in proprioceptive reweighting from age-matched asymptomatic controls (20). This discrepancy may highlight an age-related deterioration in proprioceptive reweighting of asymptomatic older adults (21). However, it remains unclear whether proprioceptive reweighting starts to deteriorate in middle-aged adults with/without non-specific CLBP. The finding may inform clinical management such as proprioceptive training to decrease the fall risk (22) or back injuries in middle-aged adults. Conscious trunk proprioception can be objectively evaluated by assessing the accuracy in repositioning of the trunk to a predetermined target position (14). Studies revealed that patients with CLBP (age range:18–74years) displayed greater repositioning errors than asymptomatic counterparts (10, 23), although contradictory findings have also been reported (24, 25). While joint reposition sense and proprioceptive reweighting reflect conscious and unconscious proprioception, respectively, no studies have evaluated whether these two aspects of proprioception are interrelated in people with and without CLBP.

Given the above, the present study aimed to: (1) compare RPW and lumbar repositioning errors in young adults with and without CLBP, as well as in middle-aged adults with and without CLBP; and (2) determine the relation between RPW and lumbar repositioning errors in young adults with and without CLBP, as well as in middle-aged adults with and without CLBP. It was hypothesized that (1) young adults with CLBP have significantly higher RPW and lumbar repositioning errors than asymptomatic counterparts, but middle-aged adults with and without CLBP will not have significant differences in RPW or lumbar repositioning errors; and (2) RPW are significantly correlated with lumbar repositioning errors in young adults with or without CLBP, and in middle-aged adults with or without CLBP.

## Materials and Methods

### Participants and Study Design

This cross-sectional study was conducted in a laboratory at a university. The study was approved by the Human Subjects Ethics Sub-committee of the university (HSEAR20151027007-01). Participants aged between 18 and 65 years with and without CLBP were recruited from a public hospital and the University campus, respectively. Participants were stratified into young (18–44 years) and middle-aged (45–65 years) subgroups to enable the within-group comparison of proprioception between those with and without CLBP. The middle-age range was chosen according to the definition documented in the 2020 report of the Lancet Commission (26).

Inclusion criteria for symptomatic participants included: (1) non-specific CLBP that required medical consultation and lasted over 3 consecutive months in the last 12 months; and (2) LBP intensity of at least 5/10 on a 11-point numeric pain rating scale (NPRS). Inclusion criteria for asymptomatic controls were no LBP at the time of visit, no history of LBP in the last 12 months, and no LBP that lasted for more than a week in the last 36 months. Exclusion criteria for all participants were history of neurological disease or vestibular impairment, systemic inflammatory disease, prior spinal surgery, neuropathy, radiculopathy, spinal infections/fractures/tumors, metabolic disorders, pregnancy, LBP conditions indicated for surgery, and red flags.

### Experimental Procedure

After providing the written informed consent, participants completed a battery of questionnaires including a questionnaire for the demographic data and history of LBP. Participants then underwent proprioception postural control tests and lumbar reposition tests.

#### Questionnaires

Pain intensity was measured using an 11-point NPRS (0 = no pain; 10 = worst pain). Participants were asked to pick a number representing the: (1) current level of pain; (2) best and worst levels of pain during the past 24 h. The average of the 3 ratings was used to estimate their level of pain over the past 24 h (27).

Hong Kong-Chinese version of Roland-Morris Disability Questionnaire was used to assess LBP-related disability (28). The 24-item questionnaire evaluates the impacts of LBP on daily function, with scores ranging from 0 to 24 (0 = no disability; 24 = maximum disability). The total score was used to classify the disability into mild (0–8), moderate (9–16), and high (17–24) severity (28). This questionnaire has demonstrated excellent reliability [intraclass correlation coefficient (ICC) = 0.94] in assessing patients with non-specific CLBP (28).

The kinesiophobia level was assessed by the 16-item Hong Kong-Chinese version of Fear Avoidance Beliefs Questionnaire (FABQ). It has shown excellent internal consistency (α = 0.8) (29), reliability and validity in measuring fear-avoidance beliefs in patients with CLBP (30). Each item was rated on a 7-point Likert-type scale (0 = completely disagree; 6 = completely agree). It comprises the Physical Activity (FABQ-PA) [4 items (2, 3, 4, 5); score range: 0–24] and the Work (FABQ-W) [7 items (6, 7, 9, 10, 11, 12, 15); score range: 0–42] subscales, while the remaining five items are excluded from calculation (30). The FABQ-PA scale is classified as low (0–14) and high fear level (15–24). The FABQ-W scale is classified as low (0–33) and high fear level (34–42) (31).

#### Proprioceptive Postural Control Test

**Figure 1:**
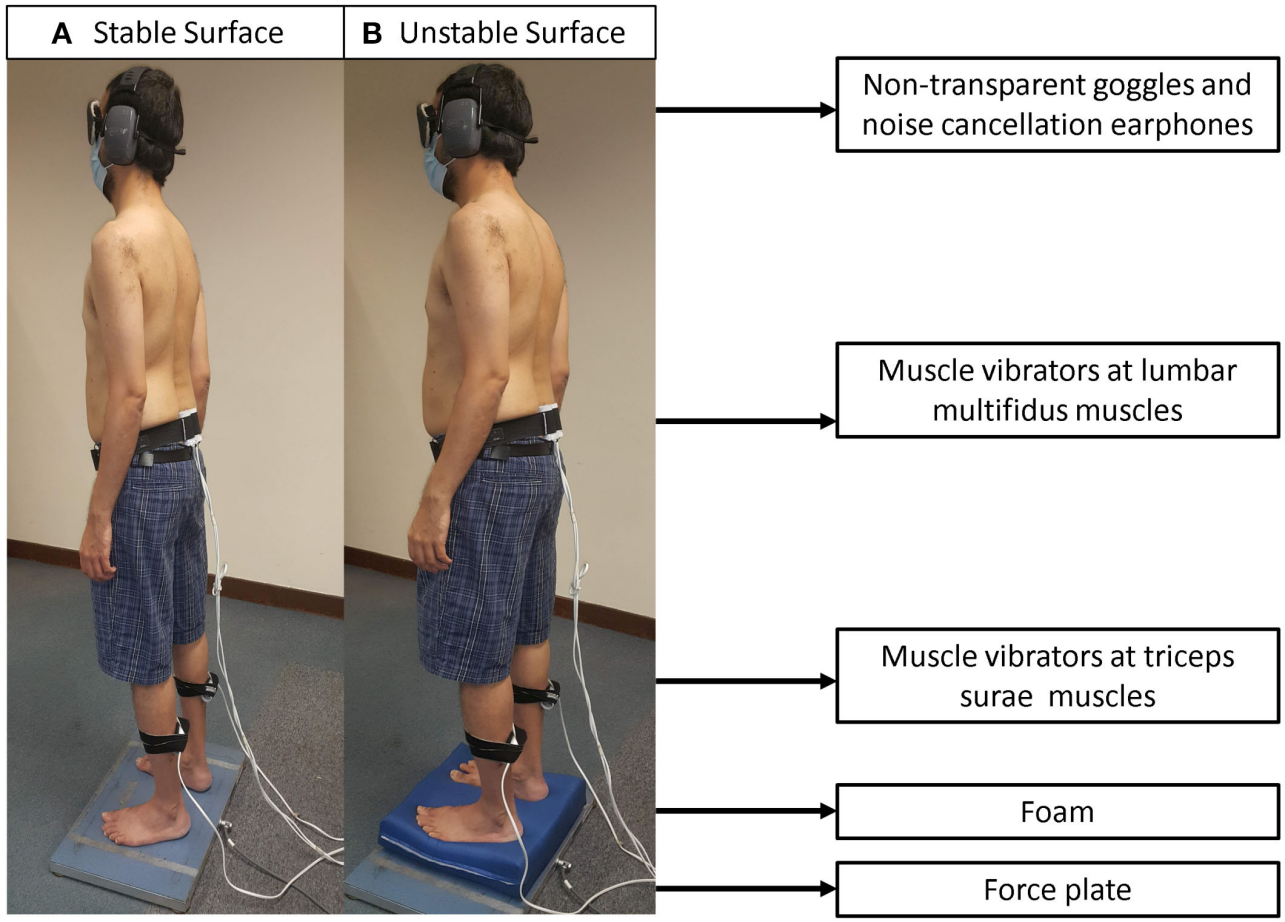
Experimental set-up: **(A)** standing on a stable surface (force plate); and **(B)** standing on an unstable surface (foam) with application of muscle vibrators on lumbar multifidus and triceps surae muscles. The displacements of center of pressure were recorded over two periods (15 s before and 15 s during the muscle vibration).

**Figure 2:**
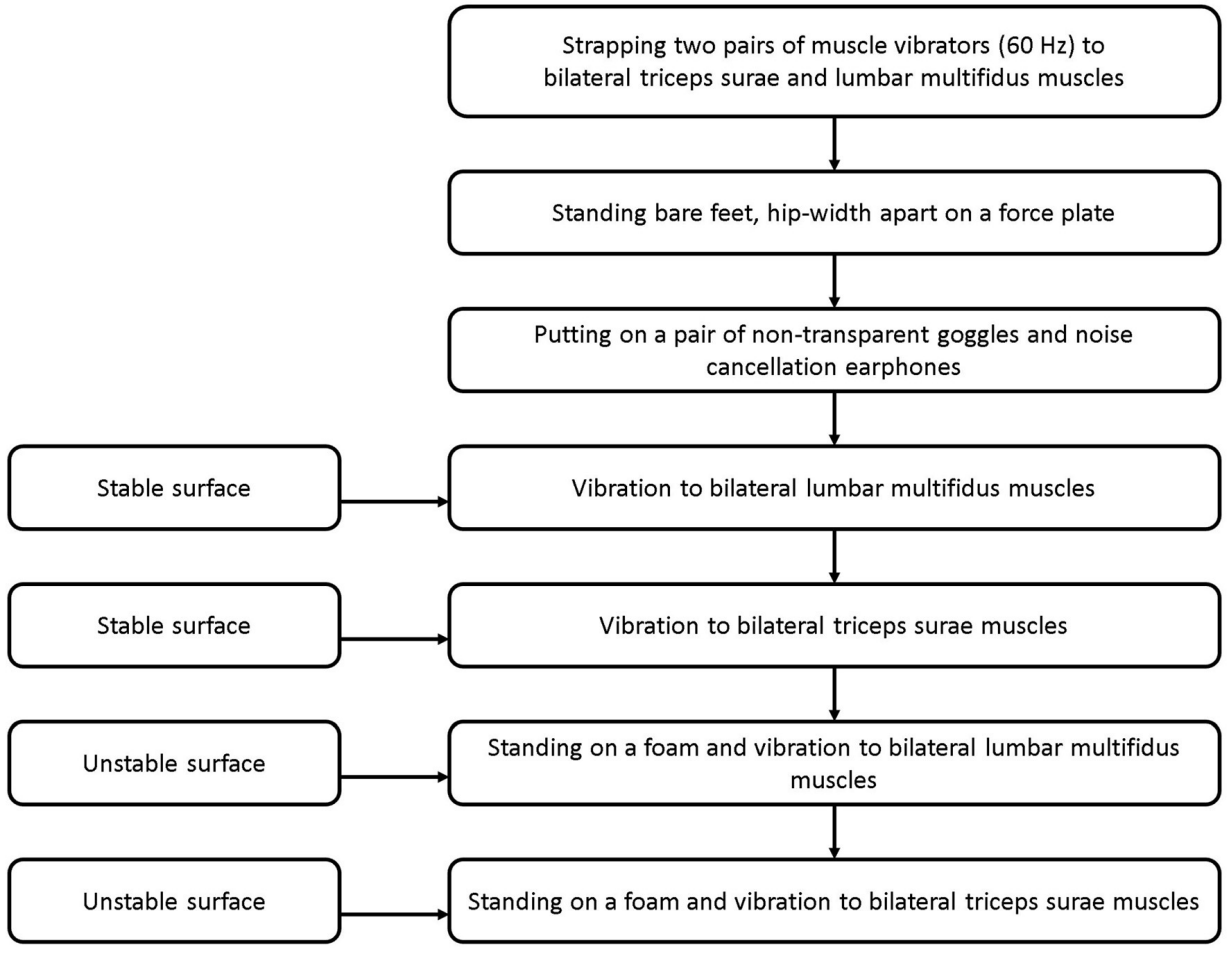
The experimental procedure for evaluating propioceptive postural control.

The RPW was evaluated using a validated force plate (32) (500 Hz, Kistler, Winterthur, Switzerland) and two pairs of muscle vibrators (60 Hz, Maxon motor Ltd., Suzhou, China) (33). Two pairs of muscle vibrators were attached bilaterally to triceps surae (TS) and lumbar multifidus muscles (LMM) at L5-S1 level, respectively. To test RPW, participants were instructed to maintain standing in an upright with bare feet about hip-width apart on a force plate, with arms hanging by the side. The participant used a pair of noise cancellation earphones to minimize noise, and goggles to occlude vision. The test comprised four standing conditions on a force plate with: (1) vibration to bilateral LMM; (2) vibration to bilateral TS; (3) a foam and vibration to bilateral LMM; (4) a foam and vibration to bilateral TS (Figures 1, 2) (33). The testing surfaces with and without a foam were considered as stable and unstable surfaces, respectively. Vibrators were used to vibrate the target muscles at an amplitude of approximately 0.5 mm. This created an illusion of muscle lengthening in the respective muscle spindles to alter proprioceptive afferents (10). The participant's center of pressure (COP) displacement data from the force plate was processed by a customized MATLAB software program (R2017a, MathWorks, Inc., Natick, MA, USA). Sagittal COP displacements were estimated using a formula: COP = Mx/Fz, where Mx is reaction moment in the sagittal plane and Fz is ground reaction force (i.e., participant's weight). The COP displacements in the trials were recorded over two periods (15 s before, and 15 s during muscle vibration) (11, 17, 33, 34).

The proprioceptive postural control strategy was estimated by RPW [RPW = absolute TS/(absolute TS + absolute LMM)], where absolute TS is the absolute value of mean sagittal COP displacement during the TS vibration trial, while absolute LMM is the absolute value of mean sagittal COP displacements during the LMM vibration trial. Higher RPW values indicate more reliance on ankle proprioceptive inputs. Conversely, lower RPW values imply increased reliance on LMM proprioceptive signals (35).

#### Lumbar Repositioning Test

The participant was instructed to sit on a stool with hips and knees at 90° flexion, and arms by the side without touching any objects. The physiotherapist identified and marked the participant's T1, T12, and S1 spinous processes and attached three electromagnetic motion sensors (MyoMotion, Noraxon, Scottsdale, AZ, USA) using a double-sided self-adhesive tape. An electromagnetic motion-tracking device (Noraxon Myomotion wireless 3D kinematic analysis system, Phoenix, USA) emits a low-frequency electromagnetic field to detect the locations of these sensors. The static and dynamic accuracy of the system is documented to be 1° and 2°, respectively, at a sampling frequency of 100 Hz (33). To collect data, an examiner guided the participant to move into and stay in a neutral sitting position for 5 s to remember the target sitting position. The participant was instructed to relax in full flexion for 5 s before reproducing the target position. The procedure was repeated thrice. No verbal feedback on the performance was given between trials. The sagittal information of the sensors during the trials was collected at 100 Hz. The data was analyzed by a customized MATLAB program to calculate the average sagittal repositioning errors with reference to the target position. The average absolute sagittal repositioning error of three measurements was calculated for data analysis.

### Statistical Analysis

Statistical analyses were performed using SPSS software (Version 22, IBM Corp., Armonk, NY). Since Shapiro-Wilk tests showed that our data was not normally distributed, non-parametric tests were used for data analysis. Data were expressed as median and inter-quartile range (IQR). Demographic variables of symptomatic and asymptomatic participants were compared by Mann-Whitney U tests (for continuous variables) or chi-square tests (for nominal variables). The significance level was set at 0.05 (2-tailed) for all tests. Effect sizes (*r*) of each observed difference were calculated by dividing the Z value by the square root of the total number of participants in that pair of groups (36). Cohen's guidelines for *r* effect sizes (0.1 = small, 0.3 = medium, 0.5 = large) were referenced (37). To determine the differential RPW characteristics of young and middle-aged adults with and without CLBP, subgroup analyses of median RPW values of people with and without CLBP in young and middle-aged subgroups (38) were performed using Mann-Whitney U tests. The relation between RPW and repositioning errors in people with and without CLBP was evaluated by Spearman's rank correlation coefficients. The strength of the correlation can be classified as very weak (0.00–0.19), weak (0.20–0.39), moderate (0.40–0.59), strong (0.60–0.79) and very strong (0.80–1.0) (39).

## Results

### Participants' Characteristics

Demographic data of 151 participants (*n* = 78 with CLBP, *n* = 73 without CLBP) is shown in Table 1. There were no significant differences in age, percentage of male, and body mass index between groups. Median age of the CLBP cohort was 46 years. Patients with CLBP demonstrated significantly higher pain intensity, disability, and FABQ scores than asymptomatic controls (*P* < 0.001). Their average LBP intensity in the last 24 h ranged from 3/10 to 6/10 on NPRS. This is reportedly due to the fluctuating pain intensity among participants with CLBP (40). Table 1 indicates that participants with CLBP had mild-moderate average pain intensity (41), mild disability (28) and significant fear avoidance beliefs (42). Similar between-group demographic results were observed in young and middle-aged subgroups except that symptomatic young adults were significantly older than asymptomatic counterparts (Table 1).

**Table 1:** Characteristics of participants with and without chronic low back pain (CLBP) [Median (interquartile range)].

**Characteristics**	**CLBP(*n* = 78)**	**Asymptomatic (*n* = 73)**	**Young (18–44 years)**		**Middle-aged (45–65 years)**	
			**CLBP (*n* = 33)**	**Asymptomatic (*n* = 31)**	**CLBP (*n* = 45)**	**Asymptomatic (*n* = 42)**
Age (year)	46.0 (35.8–54.0)	48.0 (30.0–54.5)	34.0^*^ (29.0–37.0)	29.0^*^ (23.0–34.0)	53.0 (48.5–57.5)	53.5 (45.0–64.0)
BMI (kg/m)	23.0 (21.0–25.0)	22.0 (20.0–24.0)	22.0 (20.0–25.0)	21.9 (20.0–23.0)	23.0 (21.0–25.5)	22.7 (20.7–25.0)
Gender (male %)	41.0% (32)	36.6% (26)	48.4% (16)	36.6% (11)	35.5% (16)	36.5% (15)
RMDQ	5.5^*^ (3.0–9.0)	0.0^*^ (0.0–1.0)	3.7^*^ (2.7–5.0)	0.0^*^ (0.0–1.0)	6.5^*^ (4.3–9.8)	0.0^*^ (0.0–1.0)
FABQ (0–96)	44.0^*^ (27.0–53.0)	0.0^*^ (0.0–22.0)	34.5^*^ (25.0–51.8)	0.0^*^ (0.0–21.8)	46.5^*^ (31.5–56.8)	0.0^*^ (0.0–26.0)
FABQPA (0–24)	18.0^*^ (14.0–22.0)	0.0^*^ (0.0–11.3)	20.0^*^ (15.0–21.0)	0.0^*^ (0.0–10.8)	18.0^*^ (14.3–23.0)	0.0^*^ (0.0–12.0)
FABQW (0–42)	22.0^*^ (10.0–27.0)	0.0^*^ (0.0–8.0)	19.0^*^ (8.0–29.0)	0.0^*^ (0.0–8.0)	27.0^*^ (18.0–37.0)	0.0^*^ (0.0–4.5)
Average pain intensity on NPRS (0–10)	4.2^*^ (3.0–5.6)	0.0^*^ (0.0–0.0)	3.7^*^ (2.7–3.7)	0.0^*^ (0.0–0.3)	4.5^*^ (3.4–5.9)	0.0^*^ (0.0–0.0)
Current pain intensity on NPRS (0–10)	4.0^*^ (3.0–6.0)	0.0^*^ (0.0–0.1)	3.5^*^ (3.0–5.0)	0.0^*^ (0.0–0.6)	5.0^*^ (3.3–6.0)	0.0^*^ (0.0–0.0)

*Calculation of p-values was performed using Mann-Whitney U test (for continuous variables) and chi-square test (for nominal variable). BMI, body mass index; CLBP, chronic low back pain; FABQ, fear avoidance beliefs questionnaire; FABQPA, fear avoidance beliefs questionnaire physical activity; FABQW, fear avoidance beliefs questionnaire work; NPRS, numeric pain rating scale; RMDQ, Roland Morris Disability Questionnaire*.

* *p < 0.05 for comparisons between participants with and without CLBP*.

### Proprioceptive Postural Control

**Figure 3:**
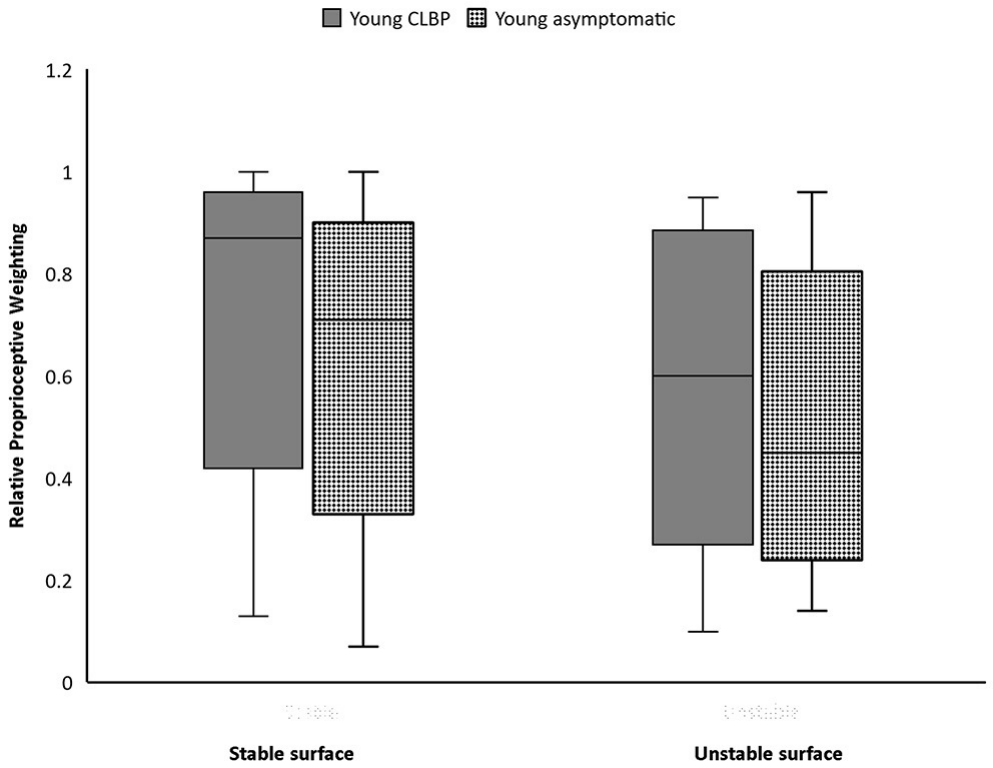
Boxplots of Relative proprioceptive weighting scores of the young people with and without chronic low back pain (CLBP).

**Figure 4:**
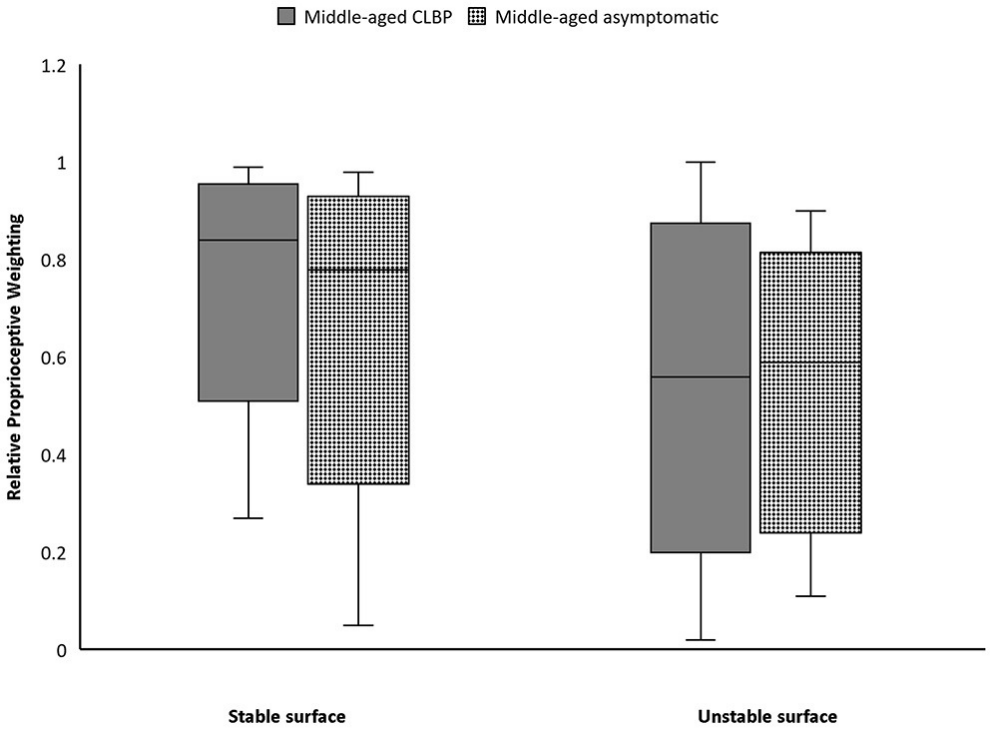
Boxplots of Relative proprioceptive weighting scores of the middle-aged people with and without chronic low back pain (CLBP).

Participants with CLBP generally demonstrated a significantly higher average RPW value than asymptomatic counterparts only on stable surface (Table 1). Subgroup analyses revealed that average RPW values of young CLBP patients were significantly higher than asymptomatic counterparts on both stable (*p* = 0.006) and unstable surfaces (*p* = 0.017) (Figure 3). While non-significant difference in RPW was noted between middle-aged adults with and without CLBP on the two testing surfaces (Figure 4, Table 2).

**Table 2:** Proprioception in people with and without chronic low back pain (CLBP) [Median (interquartile range)].

**Variables**	**CLBP**	**Asymptomatic**	***p*-value**	**Effect size**
RPW on stable surface	0.9 (0.7–0.9) (76)	0.7 (0.6–0.8) (71)	0.0^*^	−0.3
RPW on unstable surface	0.6 (0.4–0.8) (76)	0.6 (0.4–0.7) (71)	0.3	−0.1
Lumbar RE (degrees)	2.0 (0.9–3.6) (72)	1.4 (0.4–3.4) (71)	0.1	−0.1
**Subgroup analysis (young)**	**CLBP**	**Asymptomatic**		
RPW on stable surface	0.9 (0.7–0.9) (31)	0.7 (0.6–0.8) (29)	0.0^*^	−0.4
RPW on unstable surface	0.6 (0.4–0.8) (31)	0.5 (0.3–0.7) (29)	0.0^*^	−0.3
Lumbar RE (degrees)	2.0 (0.9–3.5) (29)	1.3 (0.3–3.8) (30)	0.2	−0.2
**Subgroup analysis (middle–aged)**	**CLBP**	**Asymptomatic**		
RPW on stable surface	0.8 (0.8–0.9) (45)	0.8 (0.6–0.9) (41)	0.1	−0.2
RPW on unstable surface	0.6 (0.4–0.8) (45)	0.6 (0.4–0.7) (40)	0.8	−0.0
Lumbar RE (degrees)	2.0 (0.7–3.7) (43)	1.7 (0.6–3.4) (41)	0.4	−0.1

Calculation of p-values was performed using Mann-Whitney U test. Effect sizes (r) of each observed difference were calculated by dividing the Z value by the square root of the total number of participants in that pair of groups. Cohen's guidelines for r effect sizes were used to interpret the result (0.1, small; 0.3, medium; 0.5, large). RPW, relative proprioceptive weighting; RE, repositioning error

* *p < 0.05*.

### Lumbar Repositioning Test

**Figure 5:**
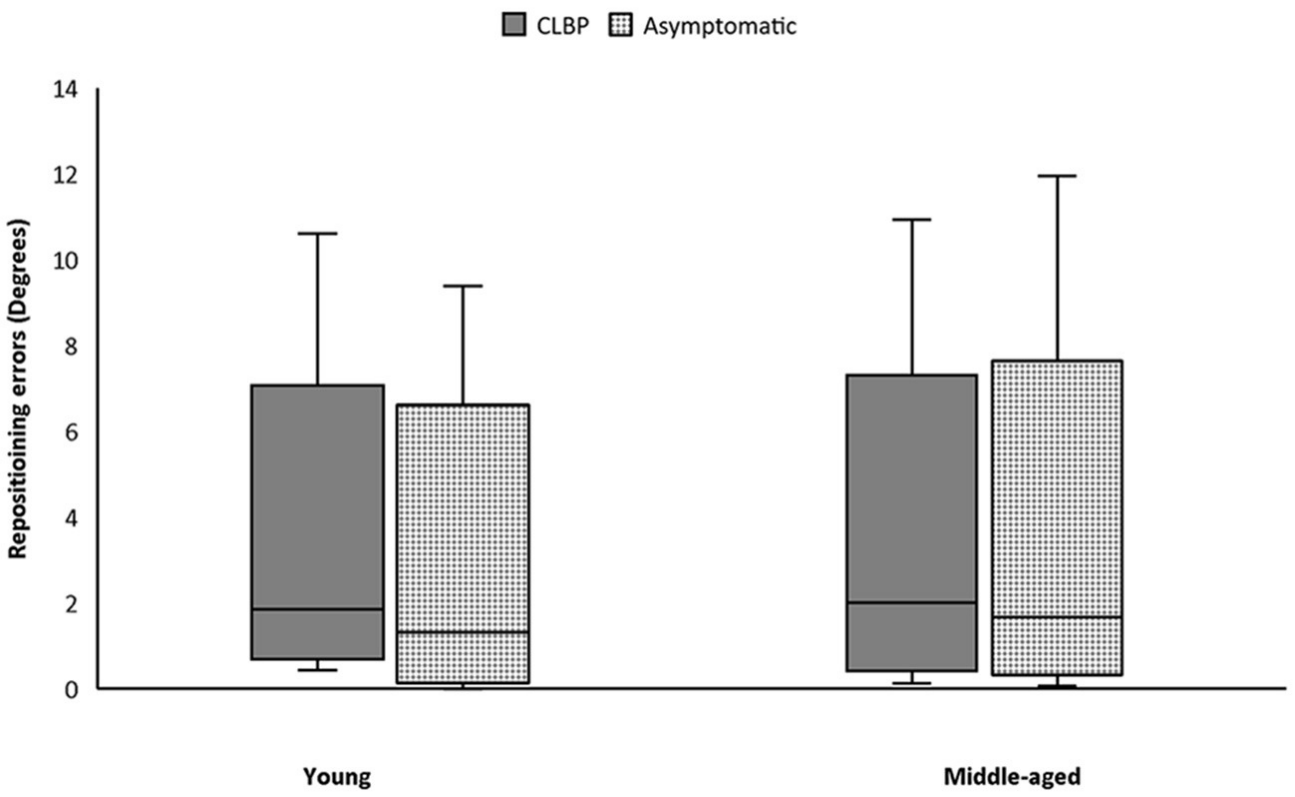
Boxplots of reposition data of young and middle-aged participants. CLBP, chronic low back pain.

Absolute mean repositioning error in patients with CLBP was larger than among asymptomatic counterparts in the whole cohort and in both subgroups. There were no significant differences in average lumbar repositioning errors between people with and without CLBP in both subgroups (Figure 5, Table 2). Additionally, there was no significant correlation between lumbar repositioning errors and RPW in people with and without CLBP in both age subgroups under both stable and unstable surface conditions (Table 3).

**Table 3:** Correlations between lumbar repositioning errors (REs) and relative proprioceptive weighting (RPW) in people with chronic low back pain (CLBP) and asymptomatic controls.

	**Variables**	**Spearman's rank correlation coefficients**	***p*-value**
CLBP young (18–44 years)	RPW on stable surface and lumbar RE	−0.02	0.9
	RPW on unstable surface and lumbar RE	0.11	0.6
CLBP middle-aged (45–65 years)	RPW on stable surface and lumbar RE	−0.15	0.3
	RPW on unstable surface and lumbar RE	0.07	0.6
Asymptomatic young (18–44 years)	RPW on stable surface and lumbar RE	−0.20	0.3
	RPW on unstable surface and lumbar RE	0.02	0.9
Asymptomatic middle-aged (45–65 years)	RPW on stable surface and lumbar RE	0.20	0.2
	RPW on unstable surface and lumbar RE	0.67	0.1

*Calculation of p-values and correlation coefficients was performed using Spearman rank correlation test. The Spearman correlation coefficient values can range from +1 to −1 where +1 indicates a perfect positive association of ranks, 0 indicates no association between ranks and −1 indicates perfect negative association of ranks. The strength of the correlation can be classified as very weak (0.00–0.19), weak (0.20–0.39), moderate (0.40–0.59), strong (0.60–0.79), and very strong (0.80–1.0)*.

## Discussion

This is the first study to examine conscious and unconscious proprioception in middle-aged people with and without CLBP. Both young and middle-aged adults with CLBP had significantly higher LBP-related disability levels and fear-avoidance beliefs than their asymptomatic counterparts. While our CLBP cohort only had mild disability, they all demonstrated high FABQ-PA. Compared to asymptomatic individuals, people with CLBP generally relied more on ankle proprioception than LMM proprioception for maintaining standing balance on a stable surface without vision. As hypothesized, young adults with CLBP significantly relied more on ankle proprioception for maintaining standing balance on both stable and unstable surfaces than asymptomatic counterparts. Such phenomenon was not observed in middle-aged adults with CLBP. Interestingly, no significant differences in lumbar repositioning errors were noted between people with and without CLBP regardless of age. The magnitude of lumbar repositioning error was also unrelated to RPW in both people with and without CLBP regardless of age.

### Relative Proprioceptive Reweighting

Young adults with CLBP rely on ankle proprioception for balance and cannot change proprioceptive weighting of ankle and trunk even when signals from TS become unreliable on unstable surface. These results concur with prior research involving young adults with CLBP (age: 18.5 ± 0.5 years) (19). Acute and chronic LBP can impair LMM proprioception (14), which may persist even after pain remission (43). Activation of nociceptors may disrupt proprioceptive signals from muscle spindles leading to reduced reliance on trunk proprioceptive signals for balance control (44). Pain may also cause the reorganization of somatosensory cortex compromising the processing of proprioception signals (14). Therefore, LBP may affect joint position sense and kinesthesia in the lumbar region (10, 19, 45). This may lead to a vicious cycle of joint instability and pain (46).

Middle-aged individuals showed difficulty in proprioceptive reweighting regardless of LBP status. This phenomenon may be attributed to age-related deterioration in the neuromuscular (especially proprioception) system. Prior studies reported age-related deterioration in proprioceptive perception (e.g., joint sense or threshold of perception of joint motion) and cortical processing of proprioceptive signals in older adults (47–51). However, little is known regarding proprioception changes in middle-aged individuals. Our findings open a new avenue for hypothesis formulation and research.

Our results suggest that asymptomatic middle-aged adults with and without CLBP start to show decreased proprioception reweighting capacity on both stable and unstable surfaces. The proprioceptive deficits in asymptomatic middle-aged people may be attributed to age-related changes in peripheral and/or central nervous systems. Although no prior research has investigated age-related deterioration in LMM proprioception (peripheral level) in middle-aged adults, LMM degeneration (muscle atrophy and increased fatty infiltration) are evidenced in these people, which may also affect muscle spindles in LMM. Atrophy of LMM starts at ~50 years and accelerates after the age of 60, resulting in impaired muscle strength and function (52). Also fatty infiltration in LMM increases with age (53). Interestingly, age-related fatty infiltration affects lumbar paraspinal muscles (9–58%) more than thigh (6–25%) or calf muscles (8–24%) in individuals aged between 24 and 76 years (54). Age-related selective LMM degeneration in middle-aged adults may lead to similar age-related decreases in sensitivity and number of intrafusal muscle fibers in LMM, as well as degenerated ascending and descending pathways (55). This results in compromised proprioceptive reweighting ability in asymptomatic middle-aged adults.

Changes in CNS of middle-aged adults may also affect their proprioceptive processing. Cortical proprioceptive processing involves primary motor cortex, primary and secondary somatosensory cortices, and supplementary motor areas (56–58). Brain atrophy commences at a rate of 5% per decade after 40 years of age (59). Research has shown that cortical thinning begins after 40 years old due to cellular shrinkage and decreases in dendrite branching (60). Likewise, decreases in frontal white matter have been reported after 45 years (61), while gray matter in frontal lobes in middle-aged adults (age: 48 years; range: 41–60 years) is significantly less than younger counterparts (average age: 29 years, range: 23–40 years) (62). Additionally, proton magnetic resonance spectroscopy research demonstrates that middle-aged adults (average age: 47 ± 3 years) have significantly lower concentration of neurotransmitters (e.g., N-acetyl-aspartate, g-aminobutyric acid, and glutamate) in prefrontal and sensorimotor cortices than young adults (63). These structural and neurotransmitter changes may explain the suboptimal proprioceptive reweighting in middle-aged adults.

Interestingly, young patients with CLBP display brain changes comparable to age-related brain changes in middle-aged adults. Reduced gray matter in brainstem and somatosensory cortex have been reported in patients with CLBP (64). A magnetic resonance imaging (MRI) study reported that gray matter density in primary somatosensory cortex is decreased in people with CLBP (65), leading to reorganization of primary somatosensory cortex and impaired connection with primary motor cortex (66). This eventually affects spinal motor control (67). Decreased connectivity and neural processing in supplementary motor areas have also been reported in people with CLBP (68, 69). One study (70) found significant decreases in neurotransmitters in the primary somatosensory cortex in people with CLBP (average age: 34 ± 11 years) as compared to asymptomatic controls. Taken together, these alterations may interrupt the interconnections between primary motor cortex, as well as primary and secondary somatosensory cortex, affecting processing of proprioceptive signals. These findings suggest that young adults with CLBP may have muscle spindle dysfunction and/or alterations in CNS that is comparable to middle-aged adults. Future research is warranted to use functional MRI and electroencephalogram to investigate structural and connectivity changes in primary motor cortex, as well as primary and secondary somatosensory cortex, in relation to altered proprioceptive reweighting ability in young and middle-aged adults with and without CLBP.

### Repositioning Errors

No difference in repositioning errors between people with and without CLBP in young and middle-aged adults accords with previous research. One study performed the same repositioning test on young adults with LBP (age: 38 ± 7 years; pain intensity on visual analog scale = 54 ± 24 mm) and found no significant difference in repositioning error between people with and without CLBP (25). Although another study reported that people with CLBP (age: 40 ± 6 years; pain intensity on visual analog scale: 6.3 ± 8.2 cm) had significantly larger trunk repositioning error than age-matched asymptomatic controls (71), their testing method differed from the current study. Specifically, their participants underwent active repositioning test in the chair of an isokinetic dynamometer, with upper trunk, bilateral thighs and pelvic immobilized by straps, which might provide extra sensory feedback to improve the test results. Further, since their participants had higher pain intensity than our symptomatic participants (median NPRS score: 4.2/10, interquartile range: 3–6/10), more severe LBP may cause greater lumbar repositioning error than those with less symptoms.

### Correlation Between RPW and Repositioning Errors

The non-significant correlations between repositioning error and RPW in the current study may stem from the fact that the repositioning test is insensitive to detect conscious proprioceptive deficits (72). Since the repositioning error in the repositioning test is affected by both the proprioceptive sense and cognitive/memory function. Participants need to have good concentration and memory to remember pre-determined target position (73, 74). If participants have a distraction or poor memory, the test results will be affected. These factors might have affected the results of the reposition test.

Our results lay the foundation for future research in middle-aged people with and without LBP. Proprioception involves joint position sense, kinesthesia, movement detection threshold, and force sense. Future studies should use established motor perception threshold tests in sitting or side lying (46, 72, 75) to evaluate an individual's ability in detecting the smallest amount of axial or sagittal trunk rotation. Similarly, dynamometer can be used to measure force sense of patients with CLBP in different age subgroups (76). Future mechanistic research is warranted to determine whether the observed changes in proprioceptive reweighting of middle-aged people occur at spinal and/or supraspinal levels. Histological studies are also needed to examine if the quantity and quality of muscle spindles in LMM in middle-aged adults are associated with other LMM characteristics (e.g., atrophy/fatty infiltration).

## Limitations

This study had some limitations. First, prior research suggested that patients with LBP classified as having a flexion pattern in the O'Sullivan classification system displayed impaired lumbar proprioception (77). Our participants were not classified into different subgroups based on that classification system, which has prevented further subgroup analyses. Second, the use of a neutral position as the target position for the lumbar repositioning tests may be highly predictable and cannot detect subtle differences between individuals. Although this method has been used in previous research (78), future studies should use more challenging repositioning tasks. Third, the duration of CLBP might affect the motor control and proprioception differently but this data was not documented. Fourth, the current study only vibrated LMM and TS at 60 Hz. While this vibration frequency was commonly used in prior studies to distinguish people with and without LBP (17), different vibration frequency may stimulate different mechanoreceptors and yield different results (21). Future studies should use a range of vibration frequency to determine whether a specific set of vibration frequency is more sensitive to discern middle-aged and older people with and without LBP. Fifth, since good postural stability relies on proper integration of visual, proprioceptive and vestibular inputs in CNS (13). Dysfunctions in any of the three systems at the peripheral, spinal and/or supraspinal level(s) may affect the postural control. Although people diagnosed with vestibular impairment were excluded in the current study, it could not rule out the possibility that some middle-aged participants might have age-related changes in their vestibular system that might confound our findings. Future studies can use advanced technologies (e.g., near-infrared spectroscopy) (79) and established tests (e.g., galvanic vestibular stimulation and vestibule-ocular reflex tests, or vestibular evoked myogenic potentials) (80) to determine the mechanisms underlying the non-significant difference in RPW between middle-aged adults with and without CLBP. Sixth, the inclusion of people aged 60–65 years might have confounded the results in the middle-aged subgroup because of aging and more severe spinal degeneration. However, our sensitive analyses yielded the same results after removing people aged 60 years or older from the analyses. According to the World Health Organization, 45–65 years of age are considered as middle-age for people living in developed countries but not for those living in developing countries due to lower life expectancy in the latter (81). As such, the generalizability of our results should be interpreted with caution.

## Conclusions

This is the first study to reveal that asymptomatic middle-aged people display difficulty in proprioceptive reweighting, which is comparable to that of young and middle-aged adults with CLBP. This finding indicates that asymptomatic middle-aged adults are at risk of suboptimal spinal control, and may explain the higher prevalence of LBP in middle-aged people than younger counterparts (82). Future investigation is warranted to answer whether asymptomatic middle-aged people with more impaired proprioceptive reweighting capacity have a higher risk of developing LBP in the future. Proprioception training and spinal manipulative therapy may improve back muscle proprioception (83, 84). This warrants further investigation to determine whether a single or a combination of these interventions can improve back proprioception and symptoms in people with LBP across lifespan

## Data Availability Statement

The raw data supporting the conclusions of this article will be made available by the authors, without undue reservation.

## Ethics Statement

The studies involving human participants were reviewed and approved by Human Subjects Ethics Sub-committee of the Hong Kong Polytechnic University (HSEAR20151027007-01). The patients/participants provided their written informed consent to participate in this study. Written informed consent was obtained from the individual for the publication of any potentially identifiable images included in this article.

## Author Contributions

SP and AW: conceptualization and design of the study, recruitment of participants, investigation, data collection, and writing the original manuscript. AW: project administration. SP, AW, MP, JC, DS, and JK: analysis and interpretation of data. All authors: contributed to the article and approved the submitted version.

## Conflict of Interest

The authors declare that the research was conducted in the absence of any commercial or financial relationships that could be construed as a potential conflict of interest.
